# Supplementary material for: Simulation and Quantitative Analysis of Spatial Centromere Distribution Patterns
Source: Cells. 2025 Mar 25;14(7):491. doi: 10.3390/cells14070491 (PMC11987964; doi:10.3390/cells14070491)
Supplement: Supplementary file 1 [file cells-14-00491-s001.zip › cells-3480169-supplementary.pdf]

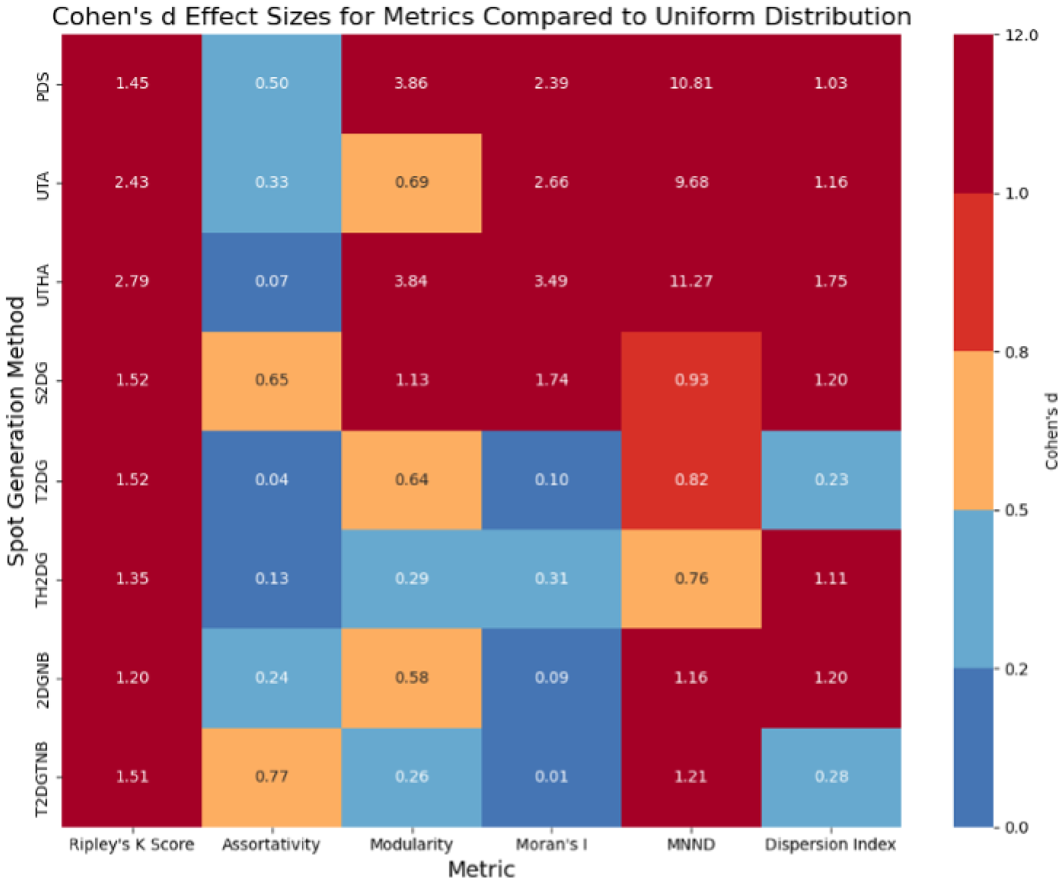

Supplementary Figure S1. Heatmap displaying Cohen's D values for clustering metrics across synthetic spatial distribution models. Metrics include Ripley's K Score, Assortativity, Modularity, Moran's I, Mean Nearest Neighbor Distance (MNND), and Dispersion Index. Cohen's D values quantify the effect size between CSR and other spatial distributions: negligible ($D<0.2$, dark blue), small ($0.2<D<0.5$, light blue), medium ($0.5 \leq D < 0.8$, orange), and large ($D \geq 0.8$, red). Ripley's K Score consistently demonstrates large effect sizes across most spatial distribution models, indicating substantial differences from CSR. MNND also shows significant differences but is sensitive to dispersion in certain models (e.g., PDS). Other metrics, such as Assortativity and Moran's I, exhibit moderate to small effect sizes for specific distributions, highlighting their limitations in detecting clustering changes robustly. These results support Ripley's K Score as a reliable metric for distinguishing centromere clustering patterns.

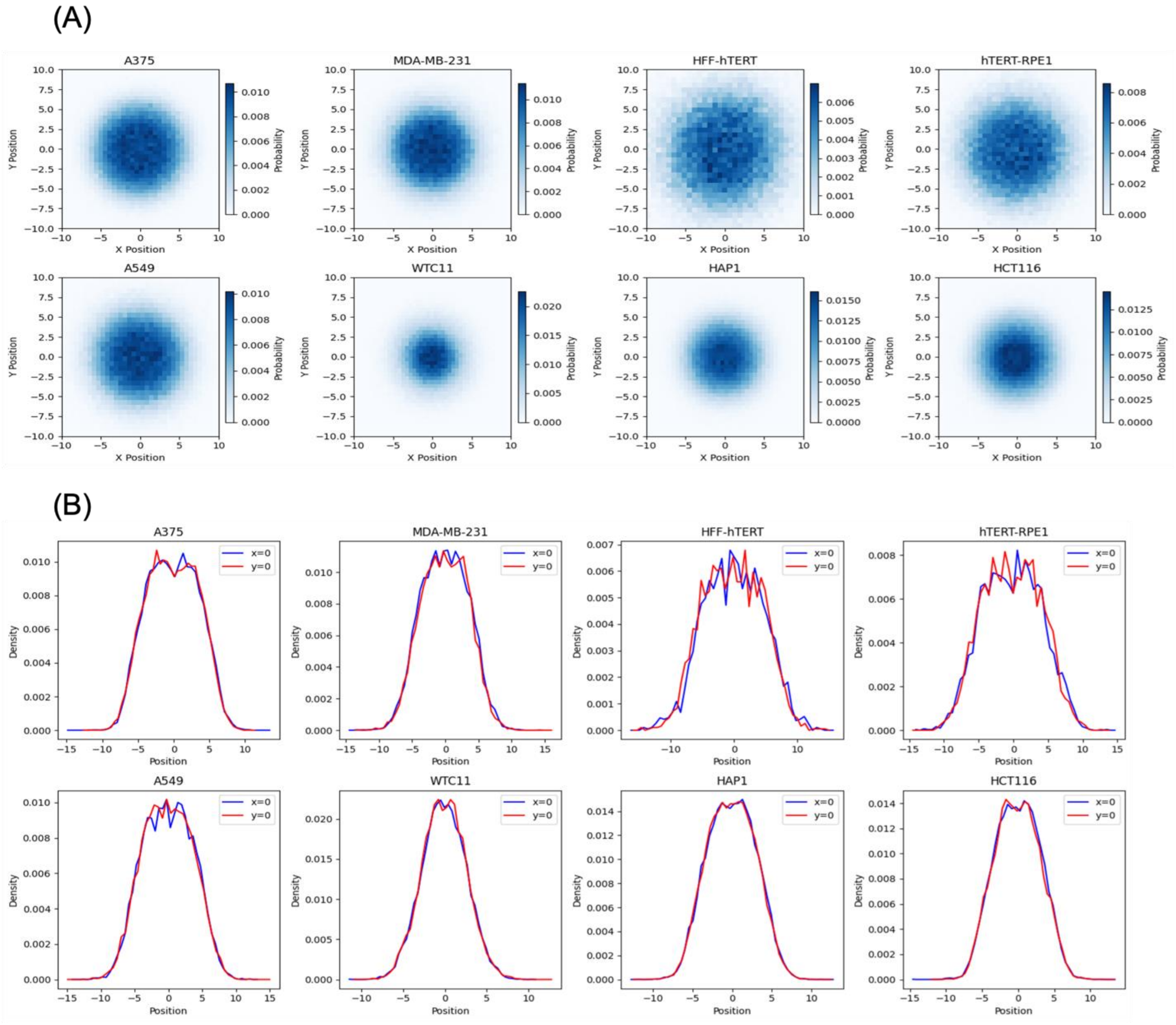

Supplementary Figure S2. Spatial distribution of standardized centromere locations across eight wild-type cell lines. (A) Overlay of nucleus-centered spot locations from HCT116 (colon), A375 (melanoma), MDA-MB-231 (breast), HFF-hTERT (fibroblast), hTERT-RPE1 (retinal), A549 (lung), HAP1 (myeloid), and WTC11 (embryonic stem cells) (A) 2D histogram showing the distribution of CENP-C spots relative to nuclear center (0,0), revealing a doughnut-shaped pattern in all cell lines. (B) Line plot analysis at x=0 and y=0 demonstrating lower spot density at the nuclear center and higher density between center and nuclear edge.

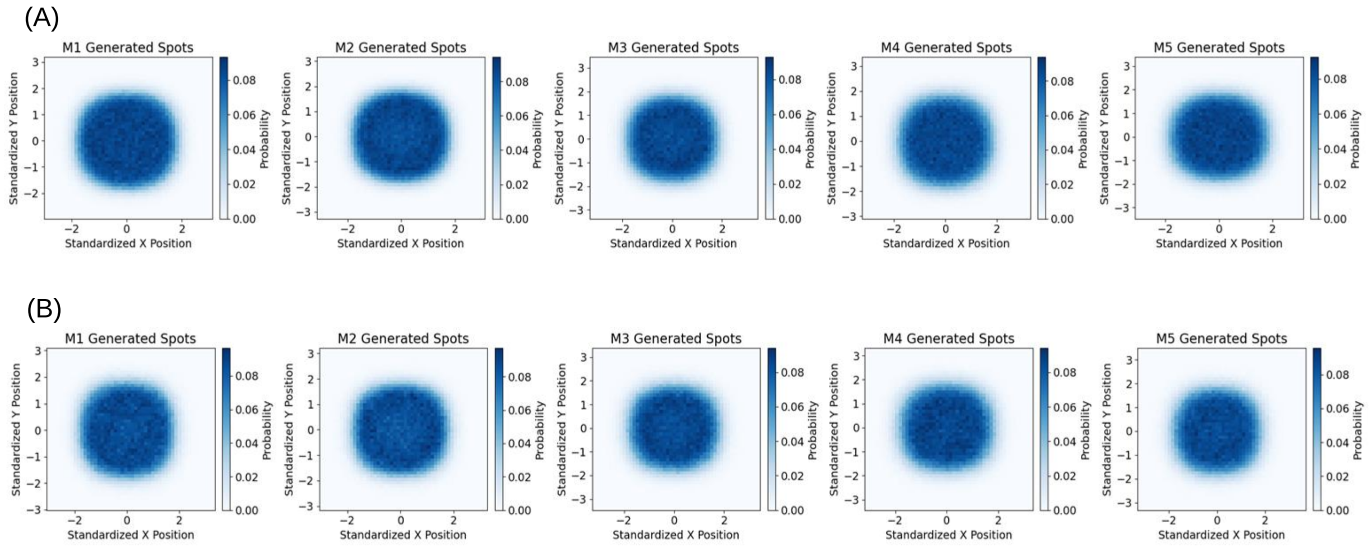

Supplementary Figure S3. Spatial distribution of standardized spot locations generated using M1-M5 methods. 2D histograms showing the standardized distribution of generated spots relative to the nuclear center (0,0), revealing a doughnut-shaped pattern on the surface of nuclei in (A) Control and (B) siNCAPH2 HCT116 cell line.

Supplementary Table S1. The sources of cell lines, culture conditions, media compositions and relevant references of respective culture protocols for the eight cell lines.

	CULTURE CONDITIONS				MEDIA COMPOSITION					
**CELL LINE**	**Split ratio**	**Temperature**	**Percent CO2**	**Percent O2**	**Medium**	**Serum**	**Substrate**	**Supplement**	**Source**	**Reference/ protocol**
WTC11	1:8	37°C	5%	Ambient	mTESR1 (STEMCELL, # 85850)	none	matrigel (Corning, #356231)	Penicillin-Streptomycin (Gibco, #15140122)	Coriell Cell Repository (#GM25256)	https://www.allencell.org/written-protocols.html
HFF-HTERT	1:8	37°C	5%	Ambient	DMEM: high glucose, GlutaMAX Supplement, pyruvate (Fisher Scientific, #10569010)	10% FBS (Gibco, #10082147)	none	Penicillin-Streptomycin (Gibco, #15140122)	Job Dekker (RRID: CVCL_VC40)	https://data.4dnucleome.org/protocols/84ee9070-e0c0-478f-9bec-7df22f3e89c2/
HTERT-RPE1 CAS9	1:8	37°C	5%	Ambient	DMEM high glucose pyruvate (Gibco, #11995065)	10% FBS (Gibco, #10082147)	none	Penicillin-Streptomycin (Gibco, #15140122)	Thomas Gonatopoulos-Pournatzis laboratory	Hart et al., Cell, 2015 PMID: 26627737
HAP1 CAS9	1:8	37°C	5%	Ambient	DMEM high glucose pyruvate (Gibco, #11995065)	10% FBS (Gibco, #10082147)	none	Penicillin-Streptomycin (Gibco, #15140122)		Horizon Discovery (HD Cas9-011)
A375 CAS9	1:8	37°C	5%	Ambient	DMEM high glucose pyruvate (Gibco, #11995065)	10% FBS (Gibco, #10082147)	none	Penicillin-Streptomycin (Gibco, #15140122)		Horizon Discovery (HD Cas9-001)
HCT116 CAS9	1:8	37°C	5%	Ambient	RPMI-1640 (ATCC, #30-2001)	10% FBS (Gibco, #10082147)	none	Penicillin-Streptomycin (Gibco, #15140122)		Hart et al., Cell, 2015 PMID: 26627737

A549 CAS9	1:8	37°C	5%	Ambient	RPMI-1640 (ATCC, #30-2001)	10% FBS (Gibco, #10082147)	none	Penicillin-Streptomycin (Gibco, #15140122)	Hart et al., Cell, 2015 PMID: 26627737
MDA-MB-231 CAS9	1:8	37°C	5%	Ambient	RPMI-1640 (ATCC, #30-2001)	10% FBS (Gibco, #10082147)	none	Penicillin-Streptomycin (Gibco, #15140122)	Horizon Discovery (HD Cas9-014)

Supplementary Table S2. Statistical testing results for pairwise Mann-Whitney U tests, corrected using the Benjamini-Hochberg False Discovery Rate (BH-FDR) method, comparing clustering metrics calculated for CSR and all other spot generation methods.

Metric	*Reference Group*	*Comparison Group*	*Stat*	*p-value*	*Corrected p-value*	*Significant*
Ripley's K Score	CSR	UTHA	19621	4.37E-303	**5.8254e-303**	TRUE
Ripley's K Score	CSR	2DGNB	625446	0	**0.0000e+00**	TRUE
Ripley's K Score	CSR	PDS	930000	5.86E-298	**6.6992e-298**	TRUE
Ripley's K Score	CSR	UTA	33428	4.85E-286	**4.8478e-286**	TRUE
Ripley's K Score	CSR	T2DGTNB	893178	0	**0.0000e+00**	TRUE
Ripley's K Score	CSR	S2DG	525861.5	0	**0.0000e+00**	TRUE
Ripley's K Score	CSR	T2DG	1055834	0	**0.0000e+00**	TRUE
Ripley's K Score	CSR	TH2DG	1206212.5	0	**0.0000e+00**	TRUE
Assortativity	CSR	UTHA	536942	0.00422625	**4.8300e-03**	TRUE
Assortativity	CSR	2DGNB	4287014	9.58E-14	**1.9162e-13**	TRUE
Assortativity	CSR	PDS	622797	1.92E-21	**5.1138e-21**	TRUE
Assortativity	CSR	UTA	593698	3.99E-13	**6.3810e-13**	TRUE
Assortativity	CSR	T2DGTNB	2756891	2.25E-121	**1.7962e-120**	TRUE
Assortativity	CSR	S2DG	3160118	2.72E-82	**1.0865e-81**	TRUE
Assortativity	CSR	T2DG	4934849	0.49622343	4.96E-01	FALSE
Assortativity	CSR	TH2DG	4542913	1.81E-06	**2.4093e-06**	TRUE
Modularity	CSR	UTHA	5385	0	**0.0000e+00**	TRUE
Modularity	CSR	2DGNB	3349885	1.47E-66	**2.3572e-66**	TRUE
Modularity	CSR	PDS	997501	0	**0.0000e+00**	TRUE
Modularity	CSR	UTA	304291	6.94E-52	**9.2591e-52**	TRUE
Modularity	CSR	T2DGTNB	4236213	1.50E-15	**1.4978e-15**	TRUE
Modularity	CSR	S2DG	8020951	1.72E-218	**4.5798e-218**	TRUE
Modularity	CSR	T2DG	6760547	1.66E-75	**3.3104e-75**	TRUE
Modularity	CSR	TH2DG	6028129	6.75E-27	**7.7162e-27**	TRUE
Moran's I	CSR	UTHA	7523	0	**0.0000e+00**	TRUE
Moran's I	CSR	2DGNB	4882722	0.22062501	2.21E-01	FALSE
Moran's I	CSR	PDS	972447	4.71E-293	**9.4114e-293**	TRUE
Moran's I	CSR	UTA	21241	7.05E-301	**1.8803e-300**	TRUE
Moran's I	CSR	T2DGTNB	5407525	2.08E-05	**2.3757e-05**	TRUE
Moran's I	CSR	S2DG	9168219	0	**0.0000e+00**	TRUE
Moran's I	CSR	T2DG	5543492	1.38E-08	**1.8349e-08**	TRUE
Moran's I	CSR	TH2DG	6712341	1.57E-71	**2.5143e-71**	TRUE
MNND	CSR	UTHA	1000000	0	**0.0000e+00**	TRUE

MNND	CSR	2DGNB	8778986	0	**0.0000e+00**	TRUE
MNND	CSR	PDS	0	0	**0.0000e+00**	TRUE
MNND	CSR	UTA	1000000	0	**0.0000e+00**	TRUE
MNND	CSR	T2DGTNB	8609522	0	**0.0000e+00**	TRUE
MNND	CSR	S2DG	8118659	1.03E-232	**1.3698e-232**	TRUE
MNND	CSR	T2DG	7673964	1.24E-171	**1.4190e-171**	TRUE
MNND	CSR	TH2DG	7388535	2.34E-137	**2.3425e-137**	TRUE
Dispersion Index	CSR	UTHA	93180	7.60E-218	**1.5205e-217**	TRUE
Dispersion Index	CSR	2DGNB	9094784	0	**0.0000e+00**	TRUE
Dispersion Index	CSR	PDS	764597	2.62E-93	**3.4939e-93**	TRUE
Dispersion Index	CSR	UTA	200042	2.33E-119	**3.7284e-119**	TRUE
Dispersion Index	CSR	T2DGTNB	4461777	1.90E-08	**2.1661e-08**	TRUE
Dispersion Index	CSR	S2DG	9583300	0	**0.0000e+00**	TRUE
Dispersion Index	CSR	T2DG	5230485	0.01607436	**1.6074e-02**	TRUE
Dispersion Index	CSR	TH2DG	8142653	2.82E-236	**7.5134e-236**	TRUE

Supplementary Table S3. Statistical testing results for all pairwise Mann-Whitney U tests, corrected using the Benjamini-Hochberg False Discovery Rate (BH-FDR) method, comparing clustering metrics across all spot generation methods.

Metric	*Group 1*	*Group 2*	*U Statistic*	*p-value*	*Effect Size (r)*	*Corrected p-value*	*Significant*
Ripley's K Score	UTHA	2DGNB	-0.2276646	1.72E-32	TRUE		
Ripley's K Score	UTHA	PDS	1000000	0	1	0	TRUE
Ripley's K Score	UTHA	CSR	980379	4.37E-303	0.960758	9.25E-303	TRUE
Ripley's K Score	UTHA	UTA	590914	1.89E-12	0.181828	2.06E-12	TRUE
Ripley's K Score	UTHA	T2DGTNB	6026681.5	7.83E-27	0.2053363	9.40E-27	TRUE
Ripley's K Score	UTHA	S2DG	2382769.5	1.58E-164	-0.5234461	2.58E-164	TRUE
Ripley's K Score	UTHA	T2DG	4271469.5	2.75E-14	-0.1457061	3.10E-14	TRUE
Ripley's K Score	UTHA	TH2DG	4603737	3.49E-05	-0.0792526	3.70E-05	TRUE
Ripley's K Score	2DGNB	PDS	9980000	0	0.996	0	TRUE
Ripley's K Score	2DGNB	CSR	9374554	0	0.8749108	0	TRUE
Ripley's K Score	2DGNB	UTA	6587986	8.76E-62	0.3175972	1.17E-61	TRUE
Ripley's K Score	2DGNB	T2DGTNB	67416891.5	0	0.34833783	0	TRUE
Ripley's K Score	2DGNB	S2DG	39053010	2.14E-158	-0.2189398	3.36E-158	TRUE
Ripley's K Score	2DGNB	T2DG	56880308	9.88E-64	0.13760616	1.37E-63	TRUE
Ripley's K Score	2DGNB	TH2DG	58612151.5	8.71E-99	0.17224303	1.25E-98	TRUE
Ripley's K Score	PDS	CSR	70000	5.86E-298	-0.86	1.17E-297	TRUE
Ripley's K Score	PDS	UTA	0	0	-1	0	TRUE
Ripley's K Score	PDS	T2DGTNB	25000	0	-0.995	0	TRUE
Ripley's K Score	PDS	S2DG	38000	0	-0.9924	0	TRUE
Ripley's K Score	PDS	T2DG	84500	0	-0.9831	0	TRUE
Ripley's K Score	PDS	TH2DG	95000	0	-0.981	0	TRUE
Ripley's K Score	CSR	UTA	33428	4.85E-286	-0.933144	9.19E-286	TRUE
Ripley's K Score	CSR	T2DGTNB	893178	0	-0.8213644	0	TRUE
Ripley's K Score	CSR	S2DG	525861.5	0	-0.8948277	0	TRUE
Ripley's K Score	CSR	T2DG	1055834	0	-0.7888332	0	TRUE
Ripley's K Score	CSR	TH2DG	1206212.5	0	-0.7587575	0	TRUE
Ripley's K Score	UTA	T2DGTNB	5339311.5	0.00039367	0.0678623	0.00039367	TRUE
Ripley's K Score	UTA	S2DG	2144236.5	1.72E-195	-0.5711527	2.94E-195	TRUE
Ripley's K Score	UTA	T2DG	3878728	1.11E-31	-0.2242544	1.38E-31	TRUE
Ripley's K Score	UTA	TH2DG	4209096	1.45E-16	-0.1581808	1.68E-16	TRUE

Ripley's K Score	T2DGTNB	S2DG	20949277.5	0	-0.5810145	0	TRUE
Ripley's K Score	T2DGTNB	T2DG	37656162.5	7.66E-201	-0.2468768	1.38E-200	TRUE
Ripley's K Score	T2DGTNB	TH2DG	40495197	6.62E-120	-0.1900961	9.93E-120	TRUE
Ripley's K Score	S2DG	T2DG	70723497	0	0.41446994	0	TRUE
Ripley's K Score	S2DG	TH2DG	71170628	0	0.42341256	0	TRUE
Ripley's K Score	T2DG	TH2DG	51683051	3.74E-05	0.03366102	3.85E-05	TRUE
Assortativity	UTHA	2DGNB	4075812	4.80E-22	-0.1848376	7.52E-22	TRUE
Assortativity	UTHA	PDS	552317	5.09E-05	0.104634	5.73E-05	TRUE
Assortativity	UTHA	CSR	463058	0.00422625	-0.073884	0.00447486	TRUE
Assortativity	UTHA	UTA	552045	5.57E-05	0.10409	6.08E-05	TRUE
Assortativity	UTHA	T2DGTNB	2792327	1.24E-117	-0.4415346	3.72E-117	TRUE
Assortativity	UTHA	S2DG	3186111	4.89E-80	-0.3627778	1.17E-79	TRUE
Assortativity	UTHA	T2DG	4581511	1.24E-05	-0.0836978	1.44E-05	TRUE
Assortativity	UTHA	TH2DG	4309395	5.48E-13	-0.138121	7.05E-13	TRUE
Assortativity	2DGNB	PDS	6965470	1.22E-93	0.393094	3.37E-93	TRUE
Assortativity	2DGNB	CSR	5712986	9.58E-14	0.1425972	1.33E-13	TRUE
Assortativity	2DGNB	UTA	6504086	1.31E-55	0.3008172	2.63E-55	TRUE
Assortativity	2DGNB	T2DGTNB	33687912	0	-0.3262418	0	TRUE
Assortativity	2DGNB	S2DG	38829160	7.74E-165	-0.2234168	3.09E-164	TRUE
Assortativity	2DGNB	T2DG	56154937	2.33E-51	0.12309874	4.41E-51	TRUE
Assortativity	2DGNB	TH2DG	52344029	9.38E-09	0.04688058	1.16E-08	TRUE
Assortativity	PDS	CSR	377203	1.92E-21	-0.245594	2.88E-21	TRUE
Assortativity	PDS	UTA	502669	0.83628363	0.005338	0.83628363	FALSE
Assortativity	PDS	T2DGTNB	1683303	6.28E-263	-0.6633394	4.52E-262	TRUE
Assortativity	PDS	S2DG	1856712	2.27E-236	-0.6286576	1.36E-235	TRUE
Assortativity	PDS	T2DG	3798414	4.00E-36	-0.2403172	6.85E-36	TRUE
Assortativity	PDS	TH2DG	3335679	1.12E-67	-0.3328642	2.52E-67	TRUE
Assortativity	CSR	UTA	593698	3.99E-13	0.187396	5.32E-13	TRUE
Assortativity	CSR	T2DGTNB	2756891	2.25E-121	-0.4486218	7.35E-121	TRUE
Assortativity	CSR	S2DG	3160118	2.72E-82	-0.3679764	6.98E-82	TRUE
Assortativity	CSR	T2DG	4934849	0.49622343	-0.0130302	0.51040125	FALSE
Assortativity	CSR	TH2DG	4542913	1.81E-06	-0.0914174	2.17E-06	TRUE
Assortativity	UTA	T2DGTNB	2259281	3.32E-180	-0.5481438	1.50E-179	TRUE
Assortativity	UTA	S2DG	2582030	1.03E-140	-0.483594	3.71E-140	TRUE
Assortativity	UTA	T2DG	4017959	1.11E-24	-0.1964082	1.81E-24	TRUE
Assortativity	UTA	TH2DG	3736667	9.44E-40	-0.2526666	1.70E-39	TRUE
Assortativity	T2DGTNB	S2DG	57083280	1.97E-67	0.1416656	4.18E-67	TRUE
Assortativity	T2DGTNB	T2DG	71287441	0	0.42574882	0	TRUE
Assortativity	T2DGTNB	TH2DG	68097303	0	0.36194606	0	TRUE
Assortativity	S2DG	T2DG	66967497	0	0.33934994	0	TRUE
Assortativity	S2DG	TH2DG	63198216	2.82E-229	0.26396432	1.45E-228	TRUE
Assortativity	T2DG	TH2DG	46330947	2.54E-19	-0.0733811	3.66E-19	TRUE
Modularity	UTHA	2DGNB	9851051	0	0.9702102	0	TRUE
Modularity	UTHA	PDS	999000	0	0.998	0	TRUE
Modularity	UTHA	CSR	994615	0	0.98923	0	TRUE
Modularity	UTHA	UTA	981028	1.02E-303	0.962056	1.54E-303	TRUE
Modularity	UTHA	T2DGTNB	9847574	0	0.9695148	0	TRUE
Modularity	UTHA	S2DG	9982295	0	0.996459	0	TRUE
Modularity	UTHA	T2DG	9973255	0	0.994651	0	TRUE
Modularity	UTHA	TH2DG	9933157	0	0.9866314	0	TRUE
Modularity	2DGNB	PDS	9974514	0	0.9949028	0	TRUE

Modularity	2DGNB	CSR	6650115	1.47E-66	0.330023	1.83E-66	TRUE
Modularity	2DGNB	UTA	4588528	1.73E-05	-0.0822944	1.73E-05	TRUE
Modularity	2DGNB	T2DGTNB	56944343	6.96E-65	0.13888686	8.36E-65	TRUE
Modularity	2DGNB	S2DG	88820027	0	0.77640054	0	TRUE
Modularity	2DGNB	T2DG	80451365	0	0.6090273	0	TRUE
Modularity	2DGNB	TH2DG	72973277	0	0.45946554	0	TRUE
Modularity	PDS	CSR	2499	0	-0.995002	0	TRUE
Modularity	PDS	UTA	4808	0	-0.990384	0	TRUE
Modularity	PDS	T2DGTNB	67138	0	-0.9865724	0	TRUE
Modularity	PDS	S2DG	830655	0	-0.833869	0	TRUE
Modularity	PDS	T2DG	162838	0	-0.9674324	0	TRUE
Modularity	PDS	TH2DG	194042	0	-0.9611916	0	TRUE
Modularity	CSR	UTA	304291	6.94E-52	-0.391418	8.06E-52	TRUE
Modularity	CSR	T2DGTNB	4236213	1.50E-15	-0.1527574	1.54E-15	TRUE
Modularity	CSR	S2DG	8020951	1.72E-218	0.6041902	2.38E-218	TRUE
Modularity	CSR	T2DG	6760547	1.66E-75	0.3521094	2.13E-75	TRUE
Modularity	CSR	TH2DG	6028129	6.75E-27	0.2056258	7.37E-27	TRUE
Modularity	UTA	T2DGTNB	6013908	3.34E-26	0.2027816	3.53E-26	TRUE
Modularity	UTA	S2DG	8933473	0	0.7866946	0	TRUE
Modularity	UTA	T2DG	8194001	5.44E-244	0.6388002	7.84E-244	TRUE
Modularity	UTA	TH2DG	7489480	4.88E-149	0.497896	6.50E-149	TRUE
Modularity	T2DGTNB	S2DG	82292048	0	0.64584096	0	TRUE
Modularity	T2DGTNB	T2DG	71936134	0	0.43872268	0	TRUE
Modularity	T2DGTNB	TH2DG	65381710	1.20970389593423e-310	0.3076342	1.8934495762449e-310	TRUE
Modularity	S2DG	T2DG	33988203	0	-0.3202359	0	TRUE
Modularity	S2DG	TH2DG	30309831	0	-0.3938034	0	TRUE
Modularity	T2DG	TH2DG	44388931	5.54E-43	-0.1122214	6.23E-43	TRUE
Moran's I	UTHA	2DGNB	9889779	0	0.9779558	0	TRUE
Moran's I	UTHA	PDS	1000000	0	1	0	TRUE
Moran's I	UTHA	CSR	992477	0	0.984954	0	TRUE
Moran's I	UTHA	UTA	624922	3.89E-22	0.249844	4.38E-22	TRUE
Moran's I	UTHA	T2DGTNB	9684578	0	0.9369156	0	TRUE
Moran's I	UTHA	S2DG	9980639	0	0.9961278	0	TRUE
Moran's I	UTHA	T2DG	9245608	0	0.8491216	0	TRUE
Moran's I	UTHA	TH2DG	9803118	0	0.9606236	0	TRUE
Moran's I	2DGNB	PDS	9804640	0	0.960928	0	TRUE
Moran's I	2DGNB	CSR	5117278	0.22062501	0.0234556	0.22062501	FALSE
Moran's I	2DGNB	UTA	292267	0	-0.9415466	0	TRUE
Moran's I	2DGNB	T2DGTNB	55279291	3.00E-38	0.10558582	3.60E-38	TRUE
Moran's I	2DGNB	S2DG	92349843	0	0.84699686	0	TRUE
Moran's I	2DGNB	T2DG	56580386	1.90E-58	0.13160772	2.36E-58	TRUE
Moran's I	2DGNB	TH2DG	68276810	0	0.3655362	0	TRUE
Moran's I	PDS	CSR	27553	4.71E-293	-0.944894	7.06E-293	TRUE
Moran's I	PDS	UTA	249	0	-0.999502	0	TRUE
Moran's I	PDS	T2DGTNB	1093483	0	-0.7813034	0	TRUE
Moran's I	PDS	S2DG	6056898	2.49E-28	0.2113796	2.90E-28	TRUE
Moran's I	PDS	T2DG	1328461	0	-0.7343078	0	TRUE
Moran's I	PDS	TH2DG	1776936	2.06E-248	-0.6446128	2.97E-248	TRUE
Moran's I	CSR	UTA	21241	7.05E-301	-0.957518	1.10E-300	TRUE
Moran's I	CSR	T2DGTNB	5407525	2.08E-05	0.081505	2.20E-05	TRUE

Moran's I	CSR	S2DG	9168219	0	0.8336438	0	TRUE
Moran's I	CSR	T2DG	5543492	1.38E-08	0.1086984	1.50E-08	TRUE
Moran's I	CSR	TH2DG	6712341	1.57E-71	0.3424682	2.02E-71	TRUE
Moran's I	UTA	T2DGTNB	9424402	0	0.8848804	0	TRUE
Moran's I	UTA	S2DG	9966535	0	0.993307	0	TRUE
Moran's I	UTA	T2DG	8987609	0	0.7975218	0	TRUE
Moran's I	UTA	TH2DG	9621954	0	0.9243908	0	TRUE
Moran's I	T2DGTNB	S2DG	86094751	0	0.72189502	0	TRUE
Moran's I	T2DGTNB	T2DG	50964452	0.01815924	0.01928904	0.01867808	TRUE
Moran's I	T2DGTNB	TH2DG	60815181	1.23E-154	0.21630362	1.70E-154	TRUE
Moran's I	S2DG	T2DG	15431184	0	-0.6913763	0	TRUE
Moran's I	S2DG	TH2DG	19978690	0	-0.6004262	0	TRUE
Moran's I	T2DG	TH2DG	58991833	1.66E-107	0.17983666	2.22E-107	TRUE
MNND	UTHA	2DGNB	149265	0	-0.970147	0	TRUE
MNND	UTHA	PDS	0	0	-1	0	TRUE
MNND	UTHA	CSR	0	0	-1	0	TRUE
MNND	UTHA	UTA	127612	7.21E-183	-0.744776	9.61E-183	TRUE
MNND	UTHA	T2DGTNB	119	0	-0.9999762	0	TRUE
MNND	UTHA	S2DG	242157	0	-0.9515686	0	TRUE
MNND	UTHA	T2DG	3692	0	-0.9992616	0	TRUE
MNND	UTHA	TH2DG	110	0	-0.999978	0	TRUE
MNND	2DGNB	PDS	0	0	-1	0	TRUE
MNND	2DGNB	CSR	1221014	0	-0.7557972	0	TRUE
MNND	2DGNB	UTA	9419806	0	0.8839612	0	TRUE
MNND	2DGNB	T2DGTNB	43644175	1.20E-54	-0.1271165	1.31E-54	TRUE
MNND	2DGNB	S2DG	44105464	2.97E-47	-0.1178907	3.15E-47	TRUE
MNND	2DGNB	T2DG	36070986	3.97E-255	-0.2785803	5.96E-255	TRUE
MNND	2DGNB	TH2DG	32455423	0	-0.3508915	0	TRUE
MNND	PDS	CSR	1000000	0	1	0	TRUE
MNND	PDS	UTA	1000000	0	1	0	TRUE
MNND	PDS	T2DGTNB	10000000	0	1	0	TRUE
MNND	PDS	S2DG	10000000	0	1	0	TRUE
MNND	PDS	T2DG	10000000	0	1	0	TRUE
MNND	PDS	TH2DG	10000000	0	1	0	TRUE
MNND	CSR	UTA	1000000	0	1	0	TRUE
MNND	CSR	T2DGTNB	8609522	0	0.7219044	0	TRUE
MNND	CSR	S2DG	8118659	1.03E-232	0.6237318	1.48E-232	TRUE
MNND	CSR	T2DG	7673964	1.24E-171	0.5347928	1.60E-171	TRUE
MNND	CSR	TH2DG	7388535	2.34E-137	0.477707	2.81E-137	TRUE
MNND	UTA	T2DGTNB	8438	0	-0.9983124	0	TRUE
MNND	UTA	S2DG	688918	0	-0.8622164	0	TRUE
MNND	UTA	T2DG	79466	0	-0.9841068	0	TRUE
MNND	UTA	TH2DG	6934	0	-0.9986132	0	TRUE
MNND	T2DGTNB	S2DG	49367551	0.1213487	-0.012649	0.1213487	FALSE
MNND	T2DGTNB	T2DG	41183562	1.99E-103	-0.1763288	2.31E-103	TRUE
MNND	T2DGTNB	TH2DG	36985760	5.48E-223	-0.2602848	7.59E-223	TRUE
MNND	S2DG	T2DG	42860220	1.76E-68	-0.1427956	1.98E-68	TRUE
MNND	S2DG	TH2DG	39450021	3.04E-147	-0.2109996	3.77E-147	TRUE
MNND	T2DG	TH2DG	46218550	2.00E-20	-0.075629	2.06E-20	TRUE
Dispersion Index	UTHA	2DGNB	9815711	0	0.9631422	0	TRUE
Dispersion Index	UTHA	PDS	964804	1.02E-283	0.929608	1.93E-283	TRUE

Dispersion Index	UTHA	CSR	906820	7.60E-218	0.81364	1.24E-217	TRUE
Dispersion Index	UTHA	UTA	622151	3.10E-21	0.244302	3.28E-21	TRUE
Dispersion Index	UTHA	T2DGTNB	8388625	2.31E-274	0.677725	4.17E-274	TRUE
Dispersion Index	UTHA	S2DG	9896669	0	0.9793338	0	TRUE
Dispersion Index	UTHA	T2DG	7864885	1.04E-196	0.572977	1.56E-196	TRUE
Dispersion Index	UTHA	TH2DG	9631213	0	0.9262426	0	TRUE
Dispersion Index	2DGNB	PDS	1431115	4.32E-304	-0.713777	8.63E-304	TRUE
Dispersion Index	2DGNB	CSR	905216	0	-0.8189568	0	TRUE
Dispersion Index	2DGNB	UTA	436285	0	-0.912743	0	TRUE
Dispersion Index	2DGNB	T2DGTNB	8779888	0	-0.8244022	0	TRUE
Dispersion Index	2DGNB	S2DG	57824460	7.19E-82	0.1564892	8.35E-82	TRUE
Dispersion Index	2DGNB	T2DG	15286671	0	-0.6942666	0	TRUE
Dispersion Index	2DGNB	TH2DG	28712747	0	-0.4257451	0	TRUE
Dispersion Index	PDS	CSR	235403	2.62E-93	-0.529194	3.14E-93	TRUE
Dispersion Index	PDS	UTA	102926	1.24E-207	-0.794148	1.94E-207	TRUE
Dispersion Index	PDS	T2DGTNB	2320486	2.45E-172	-0.5359028	3.53E-172	TRUE
Dispersion Index	PDS	S2DG	9379215	0	0.875843	0	TRUE
Dispersion Index	PDS	T2DG	3990037	5.18E-26	-0.2019926	5.65E-26	TRUE
Dispersion Index	PDS	TH2DG	7120571	1.10E-108	0.4241142	1.41E-108	TRUE
Dispersion Index	CSR	UTA	200042	2.33E-119	-0.599916	3.11E-119	TRUE
Dispersion Index	CSR	T2DGTNB	4461777	1.90E-08	-0.1076446	1.95E-08	TRUE
Dispersion Index	CSR	S2DG	9583300	0	0.91666	0	TRUE
Dispersion Index	CSR	T2DG	5230485	0.01607436	0.046097	0.01607436	TRUE
Dispersion Index	CSR	TH2DG	8142653	2.82E-236	0.6285306	4.83E-236	TRUE
Dispersion Index	UTA	T2DGTNB	7314598	4.17E-129	0.4629196	5.77E-129	TRUE
Dispersion Index	UTA	S2DG	9755644	0	0.9511288	0	TRUE
Dispersion Index	UTA	T2DG	7092548	6.97E-106	0.4185096	8.66E-106	TRUE
Dispersion Index	UTA	TH2DG	9150306	0	0.8300612	0	TRUE
Dispersion Index	T2DGTNB	S2DG	95657596	0	0.91315192	0	TRUE
Dispersion Index	T2DGTNB	T2DG	55596332	9.12E-43	0.11192664	1.03E-42	TRUE
Dispersion Index	T2DGTNB	TH2DG	82383093	0	0.64766186	0	TRUE
Dispersion Index	S2DG	T2DG	9772087	0	-0.8045583	0	TRUE
Dispersion Index	S2DG	TH2DG	20059827	0	-0.5988035	0	TRUE
Dispersion Index	T2DG	TH2DG	72223260	0	0.4444652	0	TRUE

Supplementary Table S4. The percent change calculated as average value for each metric for up to 30 spots removed from the initial 46 spots compared to the value with 46 spots.

Metric	*Distribution*	*Percent Change (%)*
Ripley's K Score	CSR	59.5567608
Ripley's K Score	PDS	inf
Ripley's K Score	UTA	12.0172256
Ripley's K Score	UTHA	3.14291737
Ripley's K Score	S2DG	2.83261342
Ripley's K Score	T2DG	1.36536147
Ripley's K Score	TH2DG	1.86411296
Ripley's K Score	2DGNB	1.59446813
Ripley's K Score	T2DGTNB	4.94364368
Assortativity	CSR	682.231385
Assortativity	PDS	49726.8791
Assortativity	UTA	44496.7239
Assortativity	UTHA	857.562303
Assortativity	S2DG	339.190031
Assortativity	T2DG	683.523035
Assortativity	TH2DG	554.275336
Assortativity	2DGNB	406.583787
Assortativity	T2DGTNB	280.373907
Modularity	CSR	52.4011886
Modularity	PDS	54.152431
Modularity	UTA	100
Modularity	UTHA	39.8715057
Modularity	S2DG	55.0145623
Modularity	T2DG	50.1452432
Modularity	TH2DG	52.453178
Modularity	2DGNB	50.1102691
Modularity	T2DGTNB	51.4916329
Moran's I	CSR	17.7412248
Moran's I	PDS	17.6846799
Moran's I	UTA	132.437197
Moran's I	UTHA	13.5630049
Moran's I	S2DG	15.6657324
Moran's I	T2DG	14.8906965
Moran's I	TH2DG	15.3107854
Moran's I	2DGNB	14.7192826
Moran's I	T2DGTNB	15.4520555
MNND	CSR	77.2792286
MNND	PDS	32.7068625
MNND	UTA	1160.51333
MNND	UTHA	273.062842
MNND	S2DG	69.7615691
MNND	T2DG	72.571224
MNND	TH2DG	72.5743327
MNND	2DGNB	75.9056292
MNND	T2DGTNB	84.1758623
Dispersion Index	CSR	2.34686669
Dispersion Index	PDS	2.25438507
Dispersion Index	UTA	38.722864
Dispersion Index	UTHA	2.30607869
Dispersion Index	S2DG	3.97306092

Dispersion Index	T2DG	2.79085127
Dispersion Index	TH2DG	2.862863
Dispersion Index	2DGNB	2.36469957
Dispersion Index	T2DGTNB	3.02788452
